# Supplementary material for: Long-Read- and Short-Read-Based Whole-Genome Sequencing Reveals the Antibiotic Resistance Pattern of Helicobacter pylori
Source: Microbiol Spectr. 2023 Apr 17;11(3):e04522-22. doi: 10.1128/spectrum.04522-22 (PMC10269496; doi:10.1128/spectrum.04522-22)
Supplement: Supplemental file 2 — Supplemental material. Download spectrum.04522-22-s0001.pdf, PDF file, 0.7 MB [file spectrum.04522-22-s0001.pdf]

Supplementary Materials

Supplementary Figure 1. Representative image of AST. The MIC values of the strains showed here for CLR and MTZ, > 256 (resistant); LVX, 0.064 (sensitive); AMX, 0.016 (sensitive); TCY, 0.125 (sensitive).

Supplementary Figure 2. Heatmap representation of ANI analysis. Results showed that the ANI values among the species and reference strain ATCC 26695 are all larger than 95%, indicating the identities of the strains is Hp.

Supplementary Table 1. MIC values and genotypes for each Hp strain to 5 antibiotics

Supplementary Table 2. Heterozygous status of rRNA

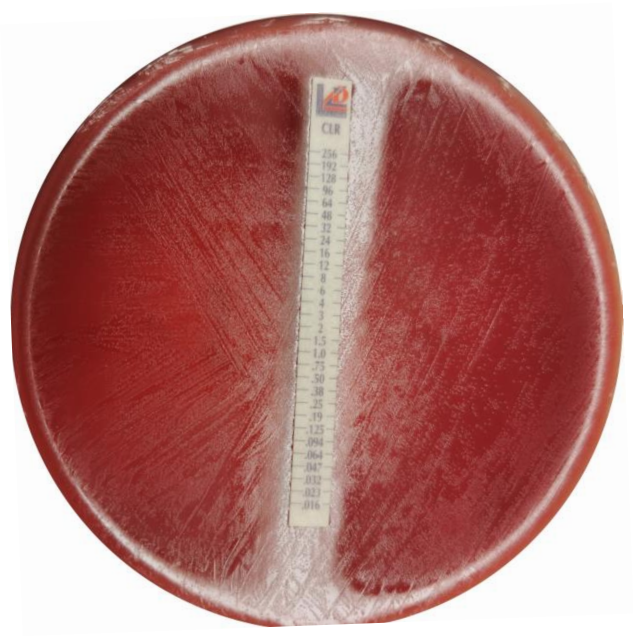

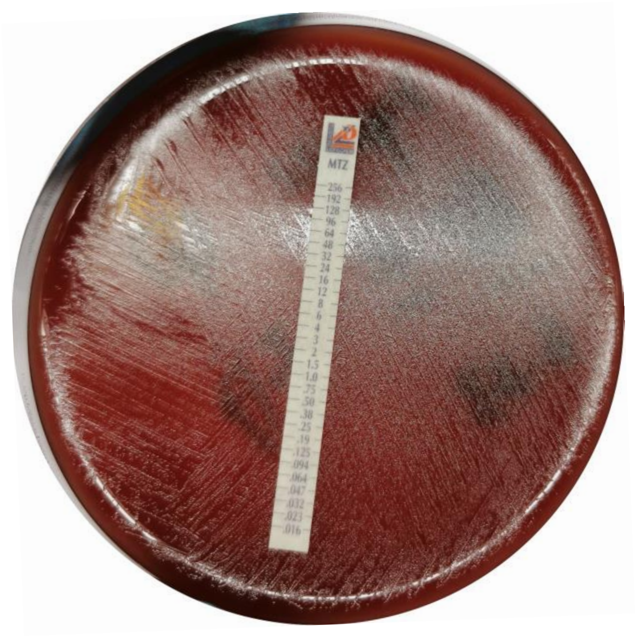

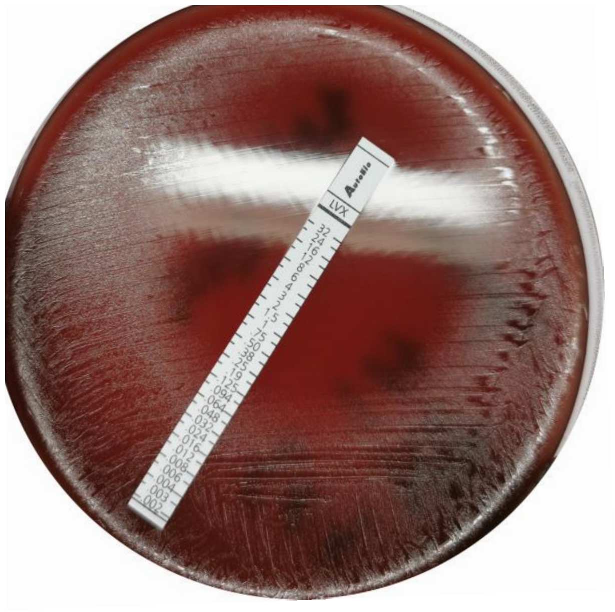

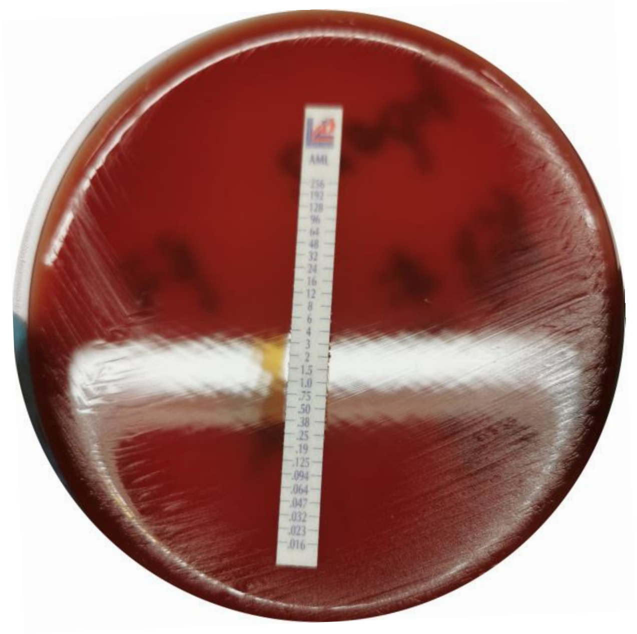

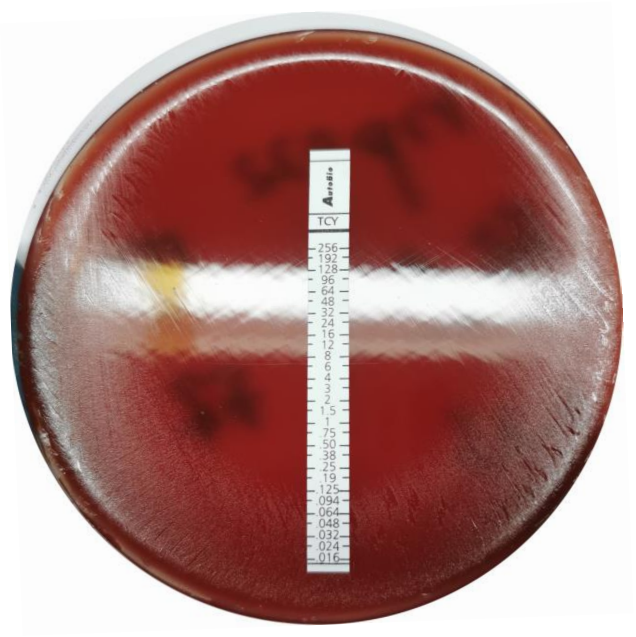

Supplementary Figure 1

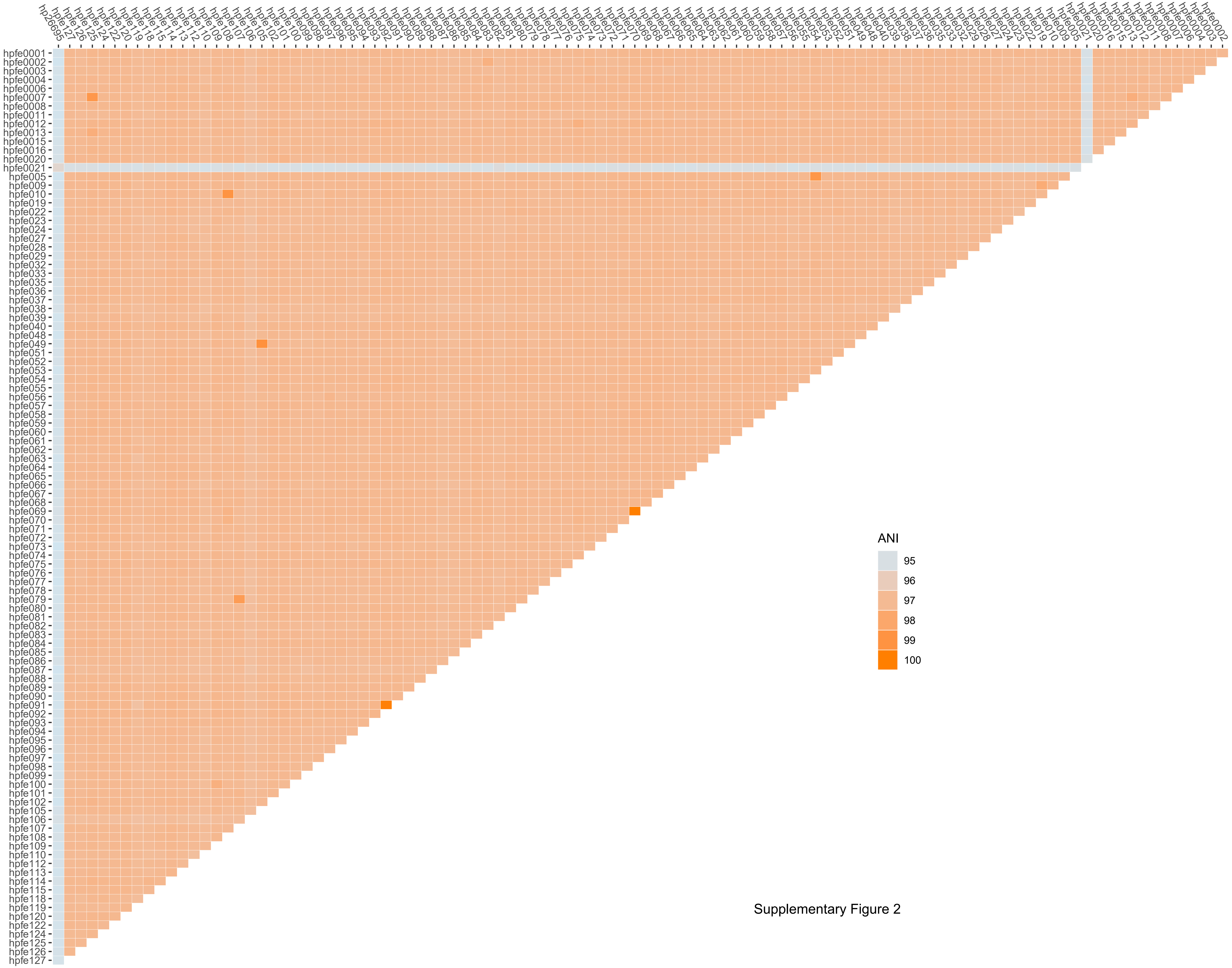

Supplementary Figure 2
